# LHX3 Interacts with Inhibitor of Histone Acetyltransferase Complex Subunits LANP and TAF-1β to Modulate Pituitary Gene Regulation

**DOI:** 10.1371/journal.pone.0068898

**Published:** 2013-07-04

**Authors:** Chad S. Hunter, Raleigh E. Malik, Frank A. Witzmann, Simon J. Rhodes

**Affiliations:** 1 Department of Biology, Indiana University-Purdue University Indianapolis, Indiana, United States of America; 2 Department of Biochemistry and Molecular Biology, Indiana School of Medicine, Indianapolis, Indiana, United States of America; 3 Department of Cellular and Integrative Physiology, Indiana University School of Medicine, Indianapolis, Indiana, United States of America; Cleveland Clinic Lerner Research Institute, United States of America

## Abstract

LIM-homeodomain 3 (LHX3) is a transcription factor required for mammalian pituitary gland and nervous system development. Human patients and animal models with *LHX3* gene mutations present with severe pediatric syndromes that feature hormone deficiencies and symptoms associated with nervous system dysfunction. The carboxyl terminus of the LHX3 protein is required for pituitary gene regulation, but the mechanism by which this domain operates is unknown. In order to better understand LHX3-dependent pituitary hormone gene transcription, we used biochemical and mass spectrometry approaches to identify and characterize proteins that interact with the LHX3 carboxyl terminus. This approach identified the LANP/pp32 and TAF-1β/SET proteins, which are components of the inhibitor of histone acetyltransferase (INHAT) multi-subunit complex that serves as a multifunctional repressor to inhibit histone acetylation and modulate chromatin structure. The protein domains of LANP and TAF-1β that interact with LHX3 were mapped using biochemical techniques. Chromatin immunoprecipitation experiments demonstrated that LANP and TAF-1β are associated with LHX3 target genes in pituitary cells, and experimental alterations of LANP and TAF-1β levels affected LHX3-mediated pituitary gene regulation. Together, these data suggest that transcriptional regulation of pituitary genes by LHX3 involves regulated interactions with the INHAT complex.

## Introduction

LIM-homeodomain (LIM-HD) class transcription factors are found in both vertebrates and invertebrates [1], [2]. These gene regulatory proteins are essential components of developmental programs in tissue and organ formation. In mammals, there are twelve genes encoding LIM-HD factors that share a similar protein domain structure featuring an amino (N-) terminus containing two LIM domains, a central homeodomain (HD), followed by a carboxyl (C-) terminus of varying length. The LIM domains are zinc-coordinated structures that mediate interactions with other proteins and intramolecular contacts that affect protein function [1]–[3]. The HD is a helical structure that forms the major DNA-binding interface. LIM-HD proteins have a characteristic HD amino acid sequence that establishes them as a specific sub-group of the large homeodomain family of transcription factors [4]. Although it is clear that LIM-HD factors play essential roles in the determination and differentiation events that guide mammalian cell type specification, little is known about the mechanisms by which LIM-HD proteins and their cofactors regulate target gene transcription.

The LIM-homeodomain 3 (or LHX3/Lim3/P-Lim) factor has critical roles in nervous system and pituitary gland development in mammals (reviewed in [5]–[8]). In the anterior pituitary, LHX3 is required for four of the five hormone-producing cell types: somatotropes, lactotropes, gonadotropes and thyrotropes [9], [10]. These specialized cells produce hormones that regulate linear growth (growth hormone [GH]), reproduction (luteinizing hormone [LH], follicle-stimulating hormone [FSH], prolactin [Prl]) and metabolism (thyroid-stimulating hormone [TSH]). In the nervous system LHX3 has defined roles in the specification of interneuron and motor neuron sub-types [11]–[14].

Inactivating mutations in the human *LHX3* gene typically lead to syndromic combined pituitary hormone deficiency diseases (CPHD) in pediatric patients that feature anterior pituitary hormone deficiencies and nervous system deficits, including deafness, developmental delay and a limited ability to rotate the neck [15]–[23]. Ablation of the mouse *Lhx3* gene also causes pituitary defects from a lack of pituitary cell differentiation [9], [10]. *Lhx3* gene knockout mice are not viable, presumably due to nervous system deficits [9], [10]. Intriguingly, an *LHX3* mutation that causes specific deletion of the C-terminus (W224ter) results in a variant form of the human disease involving pituitary hormone insufficiencies but not the deafness and neck stiffness that are correlated with LHX3 nervous system functions [19]. Similarly, a *Lhx3* W224ter mouse model is viable and recapitulates the dwarf phenotype resulting from pituitary hormone deficiencies but does not demonstrate defects linked to the nervous system [24], [25]. Further, whereas molecular studies have shown that LHX3 regulates nervous system genes within multiprotein complexes and that the N-terminal LIM domains and HD likely mediate these interactions [11], [26]–[28], pituitary gene control requires the actions of additional LHX3 protein regions, including the critical C-terminal activation and repression domains [29]–[31]. Together, these *in vitro* and *in vivo* observations are consistent with the hypothesis that the nervous system functions of LHX3 are molecularly separable in that the C-terminal part of the protein is only essential for full implementation of the pituitary roles of the protein.

LHX3 proteins *trans*-activate pituitary hormone genes, such as *alpha glycoprotein subunit* (*αGSU)* (mouse “*Cga*” gene; encoding a common subunit of FSH, LH, and TSH), the *TSH beta subunit (TSHβ/Tshb), prolactin (Prl)* and other pituitary-expressed genes (e.g. [12], [32]–[35]). LHX3 has been shown to interact with other nuclear and regulatory proteins, such as NLI/LDB, ISL1/2, PIT1, RLIM, SLB, MRG1 and CREB Binding Protein (CBP) [12], [36]–[39]. However, the role of the C-terminus in LHX3-mediated pituitary gene activation is not understood and the partners through which it exerts it functions have not been identified.

In order to further understand the role of the LHX3 C-terminus in pituitary gene transcriptional regulation, we performed an affinity purification screen to identify proteins interacting with this important region of the protein. This approach identified interactions with components of the Inhibitor of histone acetyltransferase (INHAT) multi-subunit complex. INHAT is a multifunctional repressor that inhibits histone acetylation and modulates chromatin structure (e.g. [40]–[45]). The leucine-rich acidic nuclear protein, LANP (also known as pp32, PHAP-1, mapmodulin, ANP32A and I1PP2A) and template activating factor 1β (TAF-1β/SET/PHAPII/IGAAD/I2PP2A) subunits of INHAT interact with the C-terminus of LHX3 and changes in LANP and TAF-1β levels modulate LHX3-mediated pituitary gene activation.

## Materials and Methods

### Cell Culture and Transient Transfections

Human embryonic kidney (HEK) 293T cells (1.5×10^5^ cells/35 mm dish) (from American Type Culture Collection, Manassas, VA), mouse pituitary GHFT1-5 cells (5.0×10^5^ cells/35 mm dish) (generous gift from Dr. P. Mellon, University of California San Diego; [46]) and mouse pituitary gonadotrope LβT2 cells (2.5×10^5^ cells/35 mm dish) (also from Dr. P. Mellon, University of California San Diego; [47]) were cultured and transfected as previously transcribed [31], [34], [35]. Luciferase assays were performed in triplicate and luciferase activity was measured 48 hours following transfection as previously described [31], [34], [35]. Following determination of total protein levels by the Bradford method (BioRad, Hercules, CA), luciferase activities were normalized to protein concentrations.

### Expression and Reporter Constructs-

The cloning and construction of the LHX3a and PIT1 cDNA expression plasmids into the pcDNA3 or pcDNA3.1/*Myc*-His(–)C vectors (Invitrogen, Carlsbad, CA) has been previously described [31], [34]. Use of the murine *αGSU/Cga* luciferase [48], rat *Prl* promoter [49], and murine *TSHβ* ( = *Tshb* gene) reporter [50] plasmids has been described.

### DNA Constructs

cDNAs representing the entire C-terminus of LHX3 or the C2 region of the C-terminus [31] were ligated into the pGEX-2TK protein expression plasmid (GE Healthcare, Pittsburgh, PA) as bait using the 5′-*Bam*HI and 3′-*Eco*RI cloning sites. These cDNAs were amplified by PCR with the *PfuUltra* DNA polymerase (Stratagene, La Jolla, CA). Full-length cDNAs representing human LANP, and human ataxin-3 were ligated into pGEX-2TK (or pGEX-KT). A pGEX construct expressing TAF-1β-GST was a kind gift from Dr. Ann Nardulli (University of Illinois). Full-length cDNAs for LANP (human), TAF1-β (murine), and ataxin-3 (human) were amplified and ligated into pcDNA3.1/*Myc*-His(–)C vector (Invitrogen). Additional full length plasmids containing LANP/pp32, SET/TAF1-β, and ataxin-3 cDNAs were kind gifts from Dr. Gary Pasternack (Johns Hopkins University), Dr. Ann Nardulli, and Dr. Randall Pittman (University of Pennsylvania). LANP and TAF-1β deletion constructs were generated by standard cloning procedures and sequenced to verify integrity.

### Protein Extract Preparation

Whole cell or tissue protein extracts were prepared from cultured human embryonic kidney (HEK) 293T, pituitary mouse gonadotrope LβT2 cells, or from wild type C57BL/6 mouse pituitaries as previously described [49].

### GST Fusion Protein Preparation

LHX3 bait proteins were expressed as glutathione-S-transferase (GST) fusions using the pGEX-2TK, or pGEX-KT plasmids and transforming into BL21(DE3)pLysS competent cells (Novagen, Madison, WI). Fusion protein expression was induced using isopropyl β-D-1-thiogalactopyranoside (IPTG) at 1.5 mM. Bacterial cell pellets were lysed via freeze/thaw and sonication methods, then affinity purification of fusion protein products was performed using glutathione-agarose beads (Sigma, St. Louis, MO) in MTPBS buffer [150 mM NaCl, 16 mM Na_2_HPO_4_, 4 mM NaH_2_PO_4_, plus protease inhibitor cocktails (Sigma, St. Louis, MO)]. Protein/bead complexes were washed at least four times in MTPBS plus inhibitors and a small aliquot was analyzed by SDS/PAGE electrophoresis. Acrylamide gels were stained with Coomassie Brilliant Blue to verify fusion protein purity and size.

### Affinity Purification of LHX3-interacting Proteins

Five-hundred µg of protein extracts (LβT2 or mouse pituitary extract) were first precleared at 4°C for 2 hours in the presence of glutathione bead-bound GST protein with protein interaction buffer (20 mM HEPES pH 7.9, 100 mM NaCl, 1 mM EDTA, 10% glycerol, 0.5% NP-40, 1 mM DTT, 0.5 mM PMSF, 0.2% v/v protease inhibitor cocktail (Sigma), and 50 µg/mL ethidium bromide [to inhibit nonspecific interactions possibly caused by contaminating DNA]) to reduce any nonspecific interaction of lysate proteins with the GST protein and substrates. Next, equal quantities of glutathione-GST (as a negative control) or glutathione-GST-LHX3 C-terminus were added to precleared cellular extracts (LβT2, or mouse pituitary extract) in protein interaction buffer. Following incubation, the mixes were washed with cold interaction buffer and any interacting proteins were eluted from the bait proteins using protein interaction buffer supplemented with 800 mM NaCl. Eluted proteins were precipitated with trichloroacetic acid (TCA) at a final concentration of 12.5%. Precipitated protein was resuspended in SDS reducing buffer (100 mM Tris-HCl pH 6.8, 4% SDS, 0.2% bromophenol blue, 20% glycerol, 200 mM DTT), separated using 12% acrylamide SDS-PAGE large format gels, and stained using colloidal Coomassie [20% methanol, 10% phosphoric acid, 10% ammonium sulfate, 0.12% Coomassie G250 (Sigma)]. Proteins from the GST-LHX3 C-terminus sample lane that were unique compared to the negative control GST-only lane were excised and the proteins were identified using mass spectrometry.

### Peptide Mass Fingerprinting

Proteins were excised from gels and processed automatically using a MultiProbe II Station robot (PerkinElmer). The samples were de-stained, reduced with DTT, alkylated with iodoacetamide, and tryptically digested using sequence grade, modified trypsin (Promega) in preparation for matrix-assisted laser desorption ionization mass spectrometry (MALDI-TOF MS) of the resulting peptides. The tryptic peptides were eluted and manually spotted on the sample target along with α-cyano-4-hydroxycinnamic acid matrix. The target was then analyzed directly using the prOTOF^tm^ 2000 MALDI Orthogonal Time of Flight Mass Spectrometer (PerkinElmer/SCIEX, Concord ON) using TOF Works^tm^ software for automated batch database searches of the NCBI protein sequence database. Accuracy of monoisotopic peptide mass measurements ranged between 5–15 ppm, resulting in high confidence protein identifications. Some peptide mass spectra were submitted for online interrogation of the ProFound^tm^ Peptide Mass Database. Protein identity was deemed acceptably robust, though not necessarily conclusive, when the TOF Works^tm^ expectation probability was <0.01 or the Profound^tm^ Z-score exceeded 1.30, corresponding to the 90th percentile. Proteins not identifiable by peptide mass fingerprinting and those in which post-translational modifications (phosphorylation) were subjected to LC-MS/MS using an LTQ linear ion-trap mass spectrometer (Thermo Electron). Peptide eluents prepared as described above were separated chromatographically by HPLC prior to nanoelectrospray-ionization and tandem MS. Each full scan mass spectrum was followed by three Data Dependent MS/MS spectra of the most intense peaks. The data was analyzed by Xcalibur software and the proteins were identified by the BioWorks 3.1 software suite (Thermo Electron).

### In vitro Transcription/Translation

LHX3, LHX4, LANP, TAF-1β and ataxin-3, proteins were synthesized *in vitro* from 0.5–1.0 µg pcDNA3 or pcDNA3.1-based expression vector substrates using T7 RNA polymerase, TnT rabbit reticulocyte lysates, or TnT T7 coupled wheat germ extract (Promega, Madison, WI), and cold or ^35^S-methionine or ^35^S-cysteine (Amersham, Piscataway, NJ) as previously described [31], [34].

### In vitro Protein Interaction Assays

The radiolabeled protein products were precipitated using TCA in order to determine the radionuclide incorporation. T_N_T *in vitro* translation products (2 µl) were incubated with 10 µl of BSA (10 mg/mL) and 50 µl of 0.1 M NaOH at 37°C for 15 minutes. Then 1 mL of 10% TCA was added and the mixture was incubated for an additional 15 minutes in ice. Precipitated protein was captured on GF/C glass fiber filters (Whatman) and washed with 10 ml of 5% TCA and 1 ml of acetone. The washed filters were placed in vials with 2 ml of scintillation fluid and counted. GST fusion proteins were prepared as described above. Equal quantities of GST-LHX3 and control GST alone - were added to 200,000 cpm of radiolabeled LANP, TAF-1β, or NLI as control. Interactions were brought to a final volume of 100 µl with protein interaction buffer (20 mM HEPES pH 7.9, 300 mM NaCl, 1 mM EDTA, 4 mM MgCl_2_, 0.02% NP-40, 10% glycerol, 1 mM ZnCl_2_, 1 mM DTT, 0.5 mM PMSF, and 20 µg/ml ethidium bromide) and incubated for 1 hour at 4°C with gentle rocking. After incubation, the beads were washed extensively with interaction buffer. Interacting proteins were resolved on 12% SDS-PAGE gels and detected by autoradiography.

### Western Blotting

Western analysis was performed as previously described [49]. The primary antibodies used were: anti-LHX3 polyclonal [1∶5000 (Chemicon, Temecula, CA)], mouse anti-Lim3/LHX3 monoclonal [1∶1000 (Developmental Studies Hybridoma Bank, University of Iowa, recognizes LHX3)], anti-I1PP2a (LANP/pp32) [1∶2000 (Santa Cruz Biotech, Santa Cruz, CA)], αI2PP2a (SET/TAF-1β) [1∶1000 (Santa Cruz Biotech)], anti-I2PP2a (LANP) [1∶1000 (Abcam, Cambridge, MA), anti-ataxin-3 [1∶1500 (Santa Cruz)], anti-GAPDH [1∶1000 (Santa Cruz)]. Results were visualized using SuperSignal West Dura Extended Duration Substrate (Pierce) and Biomax MR film.

### Chromatin Immunoprecipitation (ChIP)

To investigate DNA promoter occupancy by LHX3 and INHAT proteins, (non-transfected) LβT2 chromatin was prepared. Briefly, LβT2 cells (1.0×10^6^) were crosslinked with Dulbecco’s modified Eagle medium (DMEM, Invitrogen) supplemented with 1% formaldehyde and cell lysates were sonicated until cross-linked DNA was sheared to achieve lengths between 500–1000 kb. The remaining ChIP procedure was performed using the Millipore EZ-ChIP™ protocol. Relative abundance of regions of interest were measured by qPCR (ABI 7900 PRISM, Applied Biosystems, Foster City, CA) using SYBR green (Roche). Primers to the pituitary glycoprotein basic element (PGBE) region of *αGSU/Cga* promoter were as follows: *αGSU/Cga* forward primer, 5′- TCC TGT TGA AAT AAT GTA ATC CTG A-3′; *αGSU/Cga* reverse primer, 5′- TTG ACA CTT TTA AAA TAA ACA GGA CTC-3′. The percent input was calculated as 100*2^−[input-Ct(IP)]^.

### Statistical Analysis

Data points were compared using a Student’s t-test (Microsoft Excel). Values were considered significantly different when P<0.05.

## Results

### LANP and TAF-1β Interact with the LHX3 C-terminus

**Figure 1:**
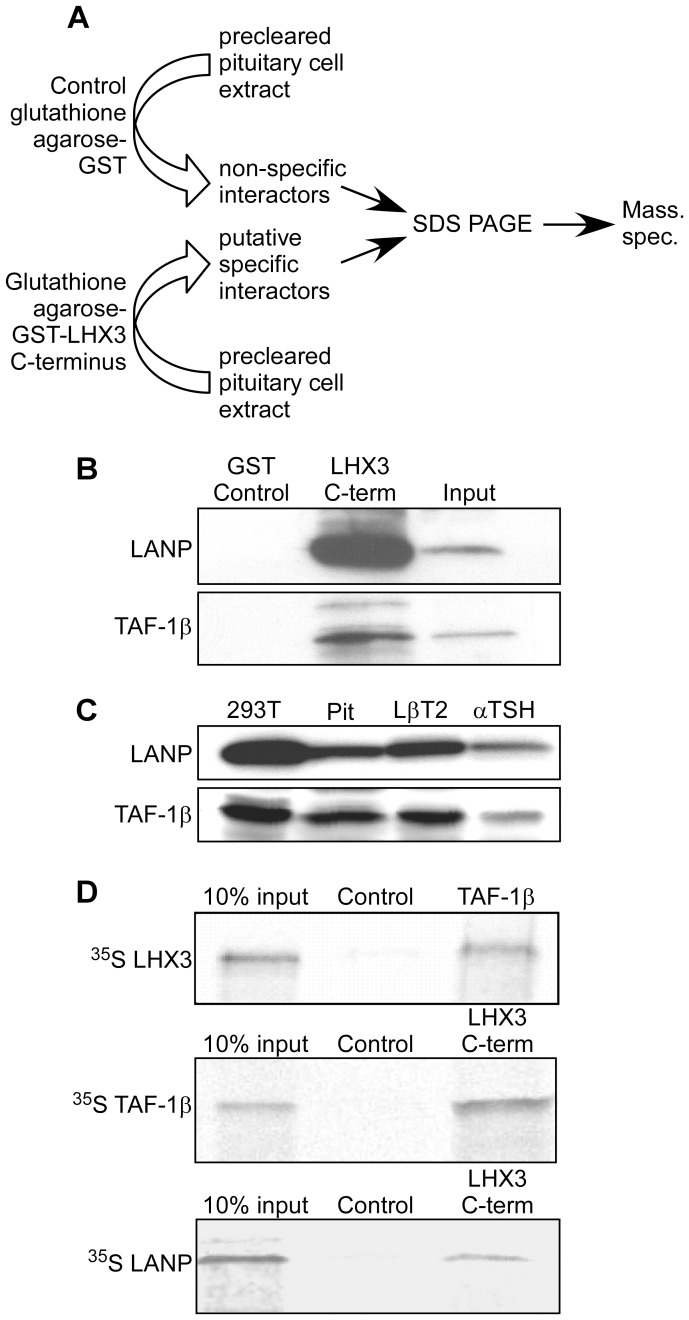
LHX3-interacting proteins isolated from pituitary cell extracts. (**A**) To identify proteins that interact with the LHX3 C-terminus, glutathione-S-transferase (GST)-human LHX3a C-terminus (residues 225–397) fusion protein was generated as “bait” and resins containing either GST-LHX3 C-terminus or GST alone (as negative control) were incubated with precleared protein extracts from cultured mouse pituitary cell lines or adult mouse pituitary gland extracts. After washing, SDS-PAGE and Coomassie staining was used to visualize proteins specifically retained by the GST-LHX3 C-terminus resins in comparison to GST negative control. Unique protein “bands” were extracted from gels and identified by mass spectrometry (mass spec.). The mass spectrometry data identified three proteins: leucine rich acidic nuclear protein (LANP), template-activating factor-1β (TAF-1β), and ataxin-3. (**B**) The interaction screens were then repeated and the recovered proteins were analyzed by western blotting. Experiments using antibodies recognizing LANP and TAF-1β confirmed enrichment for these proteins following interaction with the LHX3 C-terminus-containing resins compared to GST-alone negative control. (**C**) INHAT proteins are expressed in pituitary cells. Polyclonal antibodies against INHAT proteins were used to probe whole cell extracts of pituitary and non-pituitary cell lines. 293T = human embryonic kidney cells, Pit = pituitary lysate from pooled wild type C57/BL6 adult mouse pituitaries, LβT2 = mouse pre-gonadotrope cell line, αTSH = mouse thyrotrope cell line. (**D**) Binding of INHAT proteins to LHX3a proteins. GST-fusion resins were incubated with ^35^S radiolabeled interactor proteins in interaction buffer. After incubation, resins were washed and separated by SDS-PAGE. Gels were treated with destain/fixative then Amplify fluorography reagent (Amersham). Dried gels were exposed to Biomax MR film. Control interactions received equivalent amounts of GST alone. Similar data were obtained with full-length and C-terminal LHX3 proteins.

To identify proteins that interact with the C-terminus of LHX3, a glutathione-S-transferase (GST)-LHX3a C-terminus (human residues 225–397) fusion protein was generated as “bait” and resins containing either GST-LHX3 C-terminus or GST alone (as a negative control) were incubated with precleared protein extracts from cultured mouse pituitary cell lines or adult mouse pituitary glands (Figure 1A). After washing, SDS-PAGE followed by Coomassie staining was used to visualize eluted proteins specifically retained by the GST-LHX3 C-terminus resins in comparison to the negative controls. These proteins were extracted from gels and identified by mass spectrometry. The mass spectrometry data identified three proteins: leucine rich acidic nuclear protein (LANP), template-activating factor-1β (TAF-1β), and ataxin-3. The interaction screens were repeated and the recovered proteins were analyzed by western blotting using antibodies recognizing LANP and TAF-1β to confirm enrichment for these proteins following interaction with the LHX3 C-terminus, compared to controls (Figure 1B). Additional western blot experiments demonstrated that LANP and TAF-1β proteins are present in mouse pituitary gland extracts, mouse pituitary gonadotrope LβT2 cells, mouse pituitary thyrotrope αTSH cells, and non-pituitary human embryonic kidney (HEK) 293T cells (Figure 1C). Expression also was observed in GH-producing rat GH3 pituitary cells and the mouse pre-gonadotrope αT3 pituitary cell line (data not shown). Together, these results indicate that the LHX3 C-terminus interacts with protein complexes containing the LANP and TAF-1β proteins and that these proteins are broadly expressed in various pituitary and non-pituitary cell types.

The potential LHX3 interactions with LANP or TAF-1β were further confirmed using purified proteins and *in vitro* interaction assays. In these experiments, GST-fusion proteins were tested with radiolabeled putative partner proteins compared to GST alone as negative controls (Figure 1D). These experiments also demonstrated LHX3 interaction with LANP and TAF-1β proteins. It is important to note that the radiolabeled protein mixes used in these experiments derive from rabbit reticulocyte lysates or wheat germ extracts and other proteins are therefore present making it possible that additional proteins may be requisite components of the detected complexes.

LANP and TAF-1β are two main subunits of the Inhibitor of histone acetyltransferase (INHAT) multi-subunit complex that acts as a multifunctional repressor to inhibit histone acetylation and modulate chromatin structure (e.g. [40]–[45]). INHAT proteins have been associated with ataxin-1 (e.g. [51]) but not (to our knowledge) with ataxin-3. It is possible that ataxin-3 is another component of the INHAT complex and therefore co-purified with the LHX3 C-terminus. However, in this study ataxin-3 failed to interact with LHX3 in the *in vitro* binding assays using purified proteins (data not shown), and because LANP and TAF-1β are proteins with known chromatin regulatory functions, we focused further analyses on LANP and TAF-1β.

### LHX3 Interacts with the Acidic C-termini of INHAT Proteins LANP and TAF-1β

**Figure 2:**
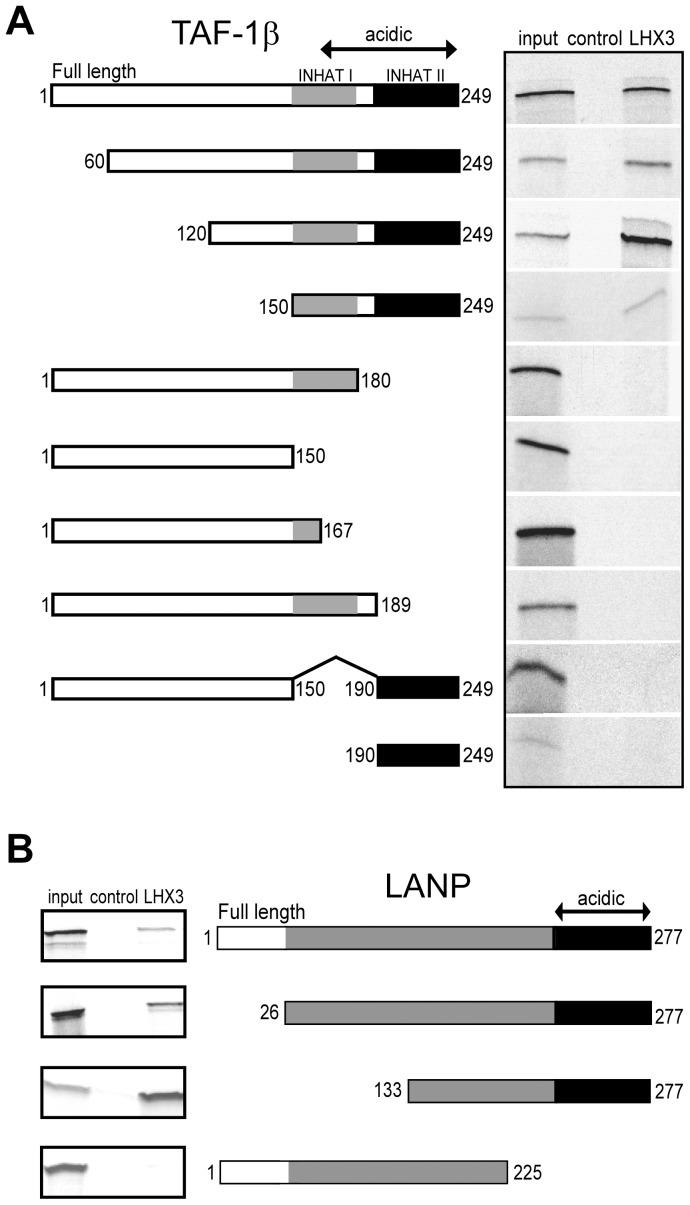
Mapping of LHX3-INHAT interactions using radiolabeled deletion constructs. (**A**) LHX3-LANP interaction was mapped using radiolabeled deletion constructs. The indicated *in vitro* translated ^35^S-labeled LANP expression constructs were incubated with either GST alone (control) or GST-LHX3 resins. Resins were washed at least four times in interaction buffer and associated proteins separated by SDS-PAGE. Gels were fixed and incubated in Amplify fluorography reagent (Amersham). Dried gels were exposed to Biomax MR film (Kodak). Input = 10% of input radiolabeled LANP lysate. (**B**) Mapping of LHX3-TAF-1β interactions using radiolabeled deletion constructs. The indicated *in vitro* translated ^35^S-labeled TAF-1β expression constructs were incubated with either GST alone (control) or GST-LHX3 resins. Reactions were washed at least four times in interaction buffer and separated by SDS-PAGE. Gels were fixed and incubated in Amplify fluorography reagent (Amersham). Dried gels were exposed to Biomax MR film (Kodak). Input = 10% of radiolabeled TAF-1β lysate.

LANP is a 249 amino acid, 28.5 kDa protein that has been ascribed with tumor suppressor protein function [52]. It has a leucine-rich N-terminus and an acidic C-terminal domain that inhibits histone acetylation [53]. To map the protein domains of LANP and TAF-1β that interact with LHX3, GST-LHX3a was incubated with radiolabeled LANP and TAF-1β protein deletion constructs (Figure 2). Full-length LANP and deletion constructs lacking N-terminal sequences (containing amino acids 1–249, 60–249, 120–249 and 150–249) were able to interact with LHX3, whereas constructs expressing LANP proteins lacking the C-terminus (amino acids 1–180, 1–150, 1–167, 1–189) or eliminating the INHAT1 domain (amino acids 150–190) failed to interact with LHX3 (Figure 2A). A small region representing the acidic LANP INHATII domain (amino acids 190–249) was not alone sufficient to interact with GST-LHX3. Thus, the acidic C-terminus of LANP, including acidic INHAT domains I and II, appears to be important for LHX3 interaction.

TAF-1β is a 277 amino acid, 39 kDa histone chaperone identified as a myeloid leukemia-associated oncoprotein [54] that belongs to the NAP-1 histone chaperone family of proteins. Like LANP, TAF-1β also has a highly acidic tail which participates in histone acetyltransferase (HAT) inhibition [40]. Thus, we hypothesized that LHX3a may also interact with TAF-1β through this domain. *In vitro* interaction assays were performed with radiolabeled TAF-1β deletion constructs (Figure 2B). Full-length TAF-1β proteins and N-terminal deletions (amino acids 26–227 and 133–277) interacted with LHX3a. However, a C-terminal deletion eliminating the acidic domain (TAF-1β 1–225) did not interact with LHX3. Together, these observations indicate that the C-terminal acidic domains of LANP and TAF-1β are required for interaction with LHX3.

### INHAT Factors are Associated with LHX3 Target Gene Promoters

**Figure 3:**
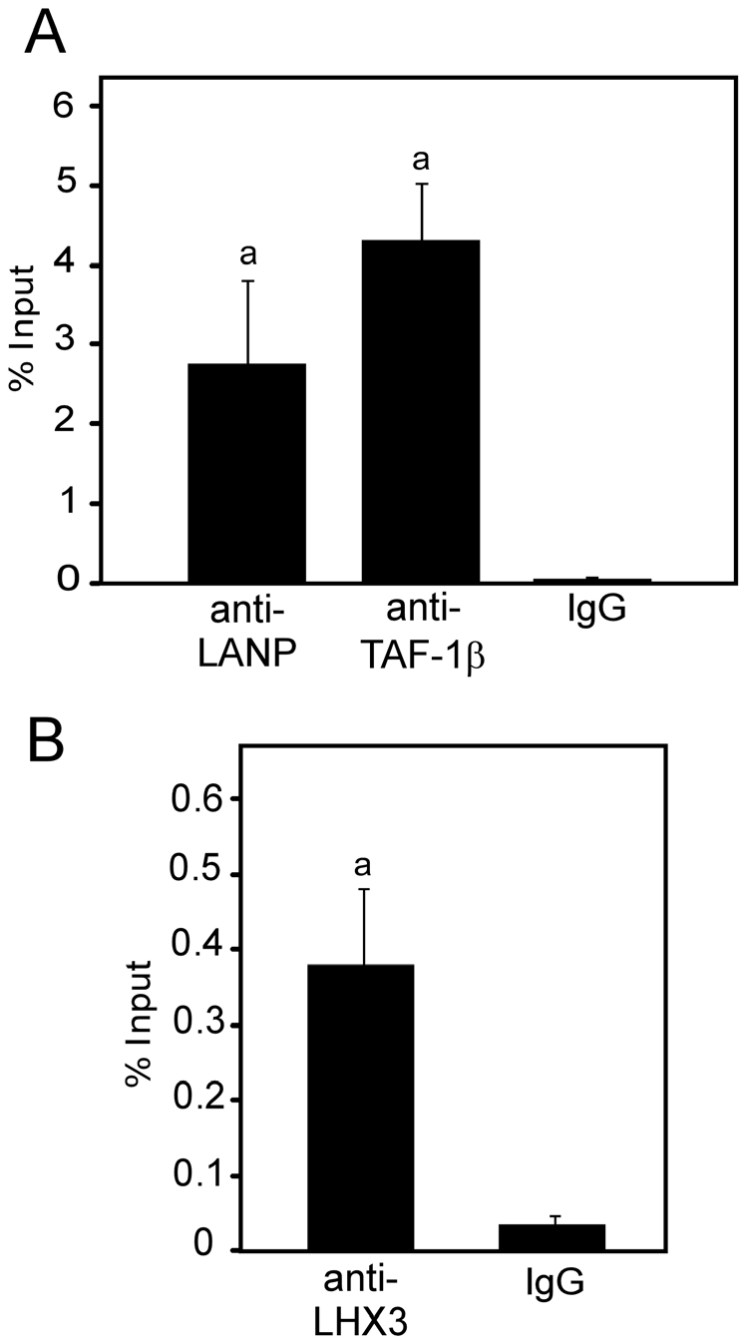
The LANP and TAF-1β INHAT proteins are associated with the LHX3-bound *αGSU/Cga* gene promoter. Chromatin immunoprecipitation (ChIP) was used to probe occupancy of the pituitary glycoprotein binding element (PGBE) region of the mouse *αGSU/Cga* promoter [12], [48] by LANP, TAF-1β (**A**) or LHX3 (**B**) proteins in LβT2 pituitary cells. ChIP enrichment was measured by quantitative PCR and represented as percent input, calculated by 100*2∧(input - Ct (IP)). Values are mean ± SEM for three independent experiments. Immunoprecipitation with non-immune species-matched IgG were carried out as negative controls. a = significantly different to the IgG control value (P<0.05).

The INHAT complex represses transcription by interacting with histones and transcriptional complexes on target promoters [55], [56]. To further investigate the role of INHAT-LHX3 complexes, experiments were performed to test if TAF-1β and LANP associate with an LHX3-responsive DNA element in a pituitary target gene promoter. Within the *alpha glycoprotein subunit (αGSU* or *Cga)* gene promoter, the pituitary glycoprotein basic element (PGBE) is a critical gene regulatory element recognized by LIM-HD proteins such as LHX3 [12]. Chromatin immunoprecipitation (ChIP) was used to assess occupation of the endogenous PGBE region of mouse *αGSU/Cga* in cultured pituitary LβT2 gonadotrope-like pituitary cells. Experiments employing antibodies recognizing LHX3, LANP and TAF-1β proteins demonstrated enrichment for PGBE-containing DNA compared to IgG and chromatin input controls (Figure 3), indicating that these proteins are associated with this key transcriptional control region. There was relatively more enrichment for PGBE-containing DNA regions in ChIP with anti-LANP and anti-TAF-1β than with anti-LHX3; this may reflect the relatively low levels of LHX3 in the LβT2 pituitary cells or differences in the properties of the available antibodies.

### INHAT Modulates LHX3-mediated Target Reporter Gene Activation

The INHAT complex has been shown to repress transcription through mechanisms including the binding of hypoacetylated histones to block acetylation by HATs and association with co-repressors including histone deacetylases (HDACs) [56], [57]. With the observed association of core INHAT proteins with LHX3 target promoter DNA, experiments were designed to assess the effects of INHAT-LHX3 interactions on known LHX3-responsive reporter genes.

**Figure 4:**
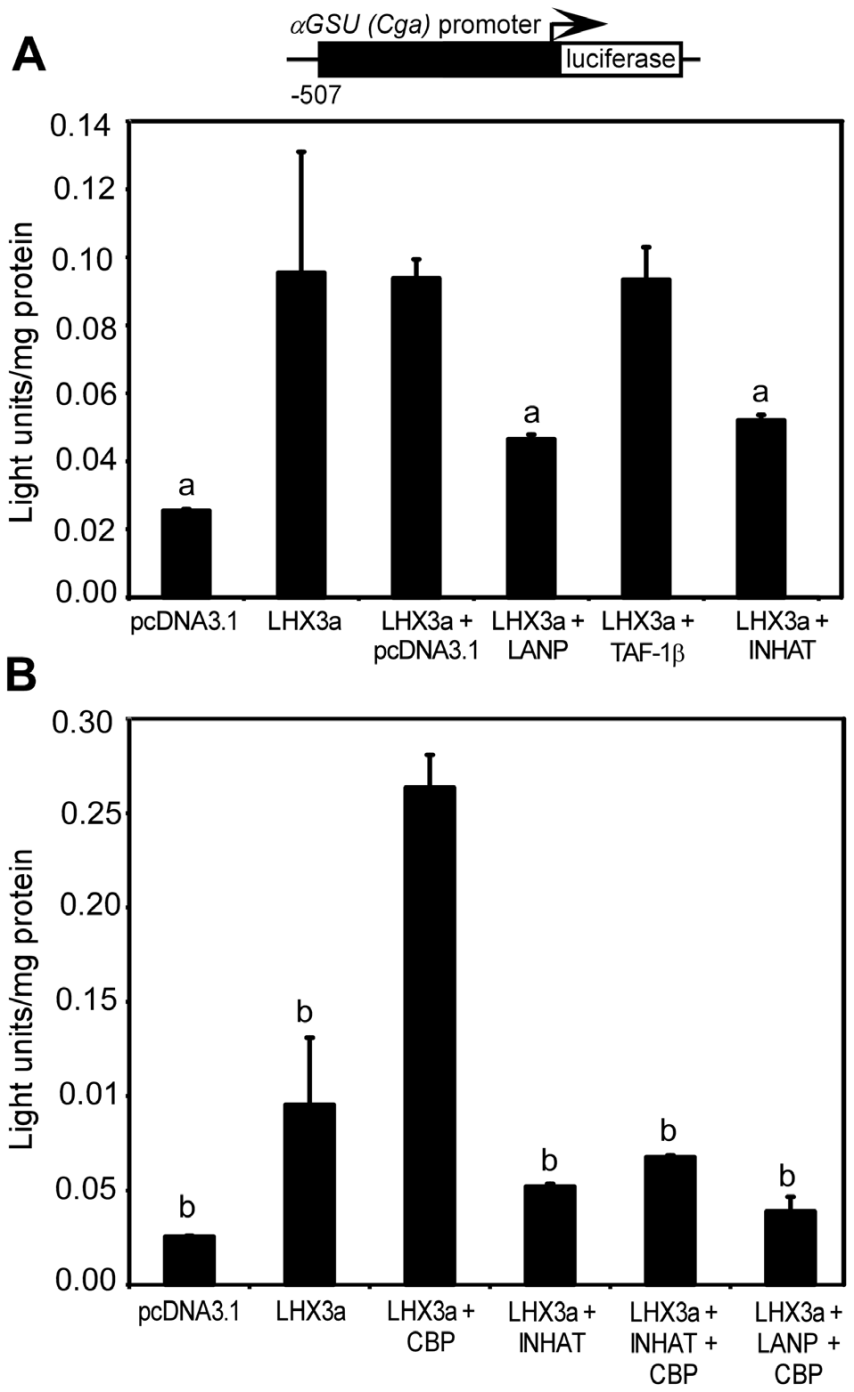
Effects of INHAT proteins on LHX3a *trans*-activation of the mouse *αGSU/Cga* promoter. (**A, B**) The indicated combinations *LHX3*, *INHAT, CBP,* or control expression vectors were transiently co-transfected into mouse pituitary GHFT1 cells with an *αGSU (Cga)* promoter *luciferase* reporter gene. Promoter activity was assayed by measurement of luciferase activity after 48 hours. Activities are mean [light units/10 seconds/µg total protein] of triplicate assays ± S.E.M. a = significantly different to the LHX3+ pcDNA3.1 value (P<0.05); b = significantly different to the LHX3+ CBP value (P<0.05).

Since INHAT proteins were associated with the *αGSU/Cga* PGBE region from ChIP analyses, we tested an *αGSU (*mouse *Cga* gene*)* promoter *luciferase* reporter gene in a reporter assay with combinations of LHX3 and INHAT proteins or control pcDNA3.1c vector transfected into pituitary GHFT1 cells. GHFT1 cells are mouse precursor pituitary cells that do not express detectable LHX3 but express the PIT1 pituitary transcription factor [46]. Compared with LHX3a *trans*-activation alone or with pcDNA3.1c vector, co-transfection of equal quantities of LHX3a and LANP reduced promoter reporter activity ∼2-fold. Additionally, co-transfection of a TAF-1β expression construct yielded similar *trans*-activation to LHX3a plus pcDNA3.1c vector. Co-transfection of the “INHAT complex” (equal quantities of SET and LANP) with LHX3a reduced *trans*-activation of the *αGSU/Cga-luciferase* reporter to similar levels as observed with co-transfection of LANP and LHX3a (Figure 4A). Additional experiments were performed using co-transfections of the INHAT components with LHX3a and the acetyltransferase CREB-binding protein (CBP) co-activator protein. Both TAF-1β and LHX3 interact with CBP presumably to modulate gene expression [58], [59]. On the *αGSU/Cga-luciferase* reporter, LHX3a and CBP are able to co-activate ∼9.4-fold over controls; however, overexpression of INHAT components reduced the *trans*-activation of the reporter to less than 25% (Figure 4B). Similar to our prior observation, LHX3-CBP co-activation also was notably reduced by LANP overexpression (Figure 4B).

**Figure 5:**
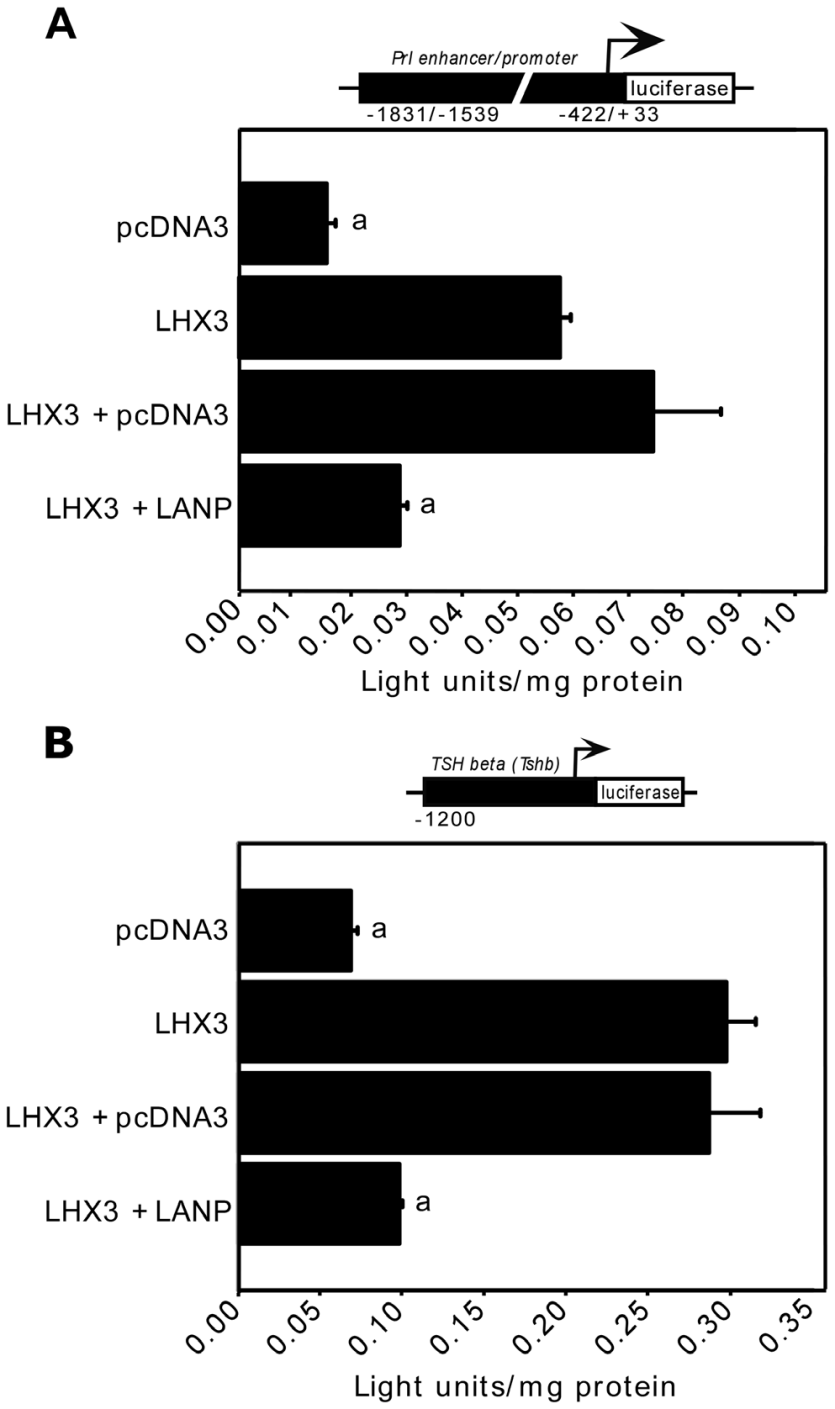
LANP represses LHX3 target promoters in pituitary GHFT1 cells. (**A**) A *Prl* enhancer/promoter *luciferase* reporter gene was transiently co-transfected into GHFT1 cells with the indicated *LHX3* and *LANP* expression vectors. a = significantly different to the LHX3+ pcDNA3.1 value (P<0.05). (**B**) The mouse *thyroid-stimulating hormone beta subunit (TSHβ/Tshb)* gene promoter reporter construct was transiently co-transfected into pituitary GHFT1 cells with the indicated *LHX3* and *LANP* expression vectors. *LHX3* and empty pcDNA3.1 vectors were co-transfected with the reporters to control for vector effects on LHX3 activity. Promoter activity was assayed by measurement of luciferase activity after 48 hours. Activities are mean [light units/10 seconds/µg total protein] of triplicate assays ± S.E.M. a = significantly different to the LHX3+ pcDNA3.1 value (P<0.05).

We then performed similar experiments using the *prolactin* gene regulatory region (enhancer/promoter; *Prl-luciferase)* and *TSHβ/Tshb* promoter *(TSHβ-luciferase)* reporter genes in pituitary GHFT1 cells. LANP also repressed LHX3-mediated transcription of both the *Prl* promoter and the *TSHβ/Tshb* promoter (Figure 5A, B).

### INHAT Modulation of LHX3-PIT1-mediated Target Reporter Gene Activation

**Figure 6:**
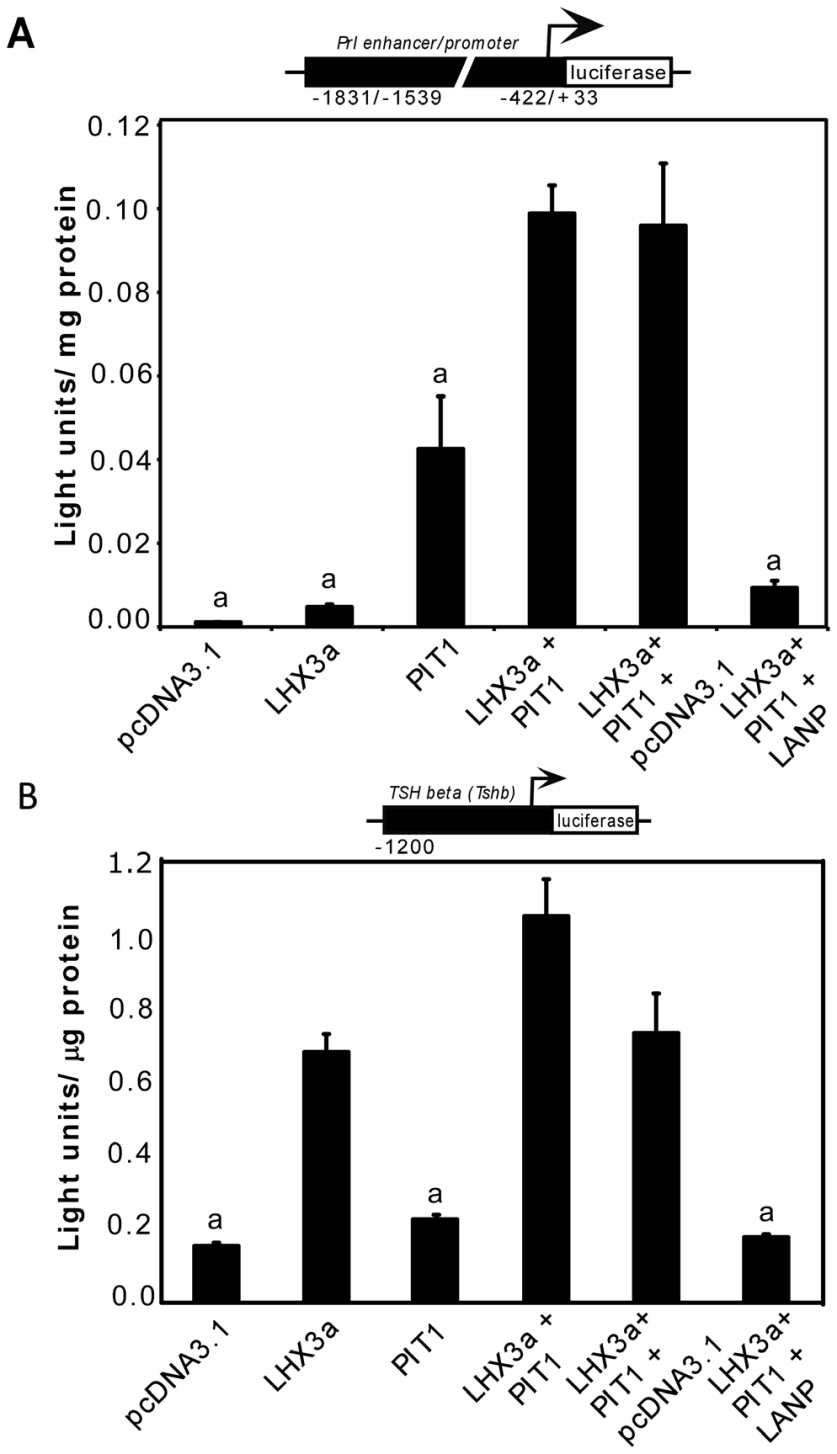
LANP represses LHX3-PIT1 synergism on target reporter genes. (**A**) LANP influence on *trans*-activation of the *Prl* promoter by LHX3 and PIT1. A *Prl* enhancer/promoter *luciferase* reporter gene was transiently transfected into 293T cells with the indicated *LHX3* and/or *PIT1* expression vectors. Promoter activity was assayed by measurement of luciferase activity after 48 hours. Activities are mean [light units/10 seconds/µg total protein] of triplicate assays ± S.E.M. WT = wild type. a = significantly different to the LHX3+ PIT1+ pcDNA3.1 value (P<0.05). (**B**) Effects of LANP on the *trans*-activation of a *luciferase* reporter gene containing the mouse *thyroid-stimulating hormone beta subunit (TSHβ/Tshb)* gene promoter by LHX3 and PIT1. The indicated combinations of *LHX3*, *PIT1*, *LANP* or control expression vectors were transiently co-transfected into 293T cells. Promoter activity was assayed by measurement of luciferase activity after 48 hours. Activities are mean [light units/10 seconds/µg total protein] of triplicate assays ± S.E.M. a = significantly different to the LHX3+ PIT1+ pcDNA3.1 value (P<0.05).

LHX3 can also activate pituitary target genes in synergy with other transcription factors, including PIT1 (e.g. [12], [34]). Experiments testing the effects of INHAT over-expression on the LHX3-PIT1 synergistic co-activation were also performed using *Prl-luciferase* and *TSHβ/Tshb-luciferase* reporters in 293T cells, a heterologous cell line that does not express PIT1 or LHX3 (Figure 6A, B). In these experiments, LANP repressed *Prl-luciferase* gene activation to less than 8%. Repression by LANP was also observed with the *TSHβ/Tshb-luciferase* reporter gene (Figure 6B).

## Discussion

LANP and TAF-1β are two main subunits of the INHAT multi-subunit complex that acts as a multifunctional repressor to inhibit histone acetylation and modulate chromatin structure (e.g. [40]–[45]). In a mechanism known as “histone masking”, INHAT binds to histone tails to prevent the substrate from interacting with histone acetyltransferases (HATs) [40]. LANP has been implicated in other processes, including neuronal differentiation, RNA shuttling, microtubule-based functions, apoptosis and inhibition of protein phosphatase 2A [60], [61]. The other principal INHAT subunit, TAF-1β, also has been associated with various functions, such as inhibition of phosphatase PP2A, apoptosis and cell cycle regulation [52], [62]. TAF-1 has been shown to be a linker histone chaperone protein involved in histone H1 dynamics [63].

The INHAT complex also modulates nuclear hormone receptor function. For example, TAF-1β and LANP repress p300-mediated acetylation of estrogen receptor α (ERα) thereby inhibiting ERα-mediated transcription [64], [65]. INHAT also interacts with the progesterone receptor and the thyroid hormone receptor beta, suggesting a role in repressing other nuclear receptor-mediated transcription. Similarly, TAF-1β interacts with the glucocorticoid receptor (GR) DNA binding domain to suppress GR-induced transcriptional activity [66]. In accord with our findings of INHAT interaction with LHX3, the observations with nuclear receptor transcription factors suggest that INHAT plays diverse and important roles in regulating gene activation.

Previous data from our laboratory suggested that the C-terminal domain of LHX3 (the amino acid sequences carboxyl to the HD – termed C1, C2, and C3) was important for overall LHX3 activity [29]–[31], [34]. The C1 domain has a nuclear localization signal (NLS), which functions in a combinatorial fashion with other NLSs to shuttle LHX3 to the nucleus, as well as conserved residues that are targets of kinases [29], [30]. The C2 domain contains the major *trans*-activation domain of LHX3 which is important for the function of the M2-LHX3 isoform, and the C3 domain, whose function is not yet described, is the most conserved domain of the LHX3 C-terminus across species [29]–[31]. Interaction of the LHX3 C-terminus with INHAT may modify activity of LHX3; most notably, our data point to a role in modulating the transcriptional activity of LHX3.

Recently, a subset of patients with CPHD was found to have an LHX3 protein lacking the C-terminus, resulting from an early termination signal at residue 224 (LHX3a W224Ter) [19]. These patients present with less severe neuroendocrine symptoms than other known human *LHX3* mutations, including delayed onset of CPHD, apparently normal pituitary morphology and a lack of the characteristic rigid neck phenotype [19]. Our lab recently developed and characterized a mouse model of the W224Ter patients, demonstrating that the C-terminus is necessary for pituitary development, but not nervous system development [24], [25]. Therefore, understanding the proteins interacting with the C-terminus provide novel insight into the role of this domain in pituitary gene activation and maintenance.

Our experiments demonstrate that LHX3 interacts with the acidic carboxyl domains of the TAF-1β and LANP INHAT components. It has been described that LANP and TAF-1β similarly interact with each other through their carboxyl terminal acidic domains [45]. INHAT components have been described as both facilitating transcriptional repression and activation via co-factor associations [56]. INHAT proteins participate in histone masking, as well as binding of target gene promoter sequences for gene repression [40], [55], [56], [67]. Our data also indicate LHX3, LANP, and TAF-1β associate with the LHX3-responsive *αGSU/Cga* promoter in mouse gonadotrope cells; and INHAT protein overexpression inhibits LHX3-driven pituitary gene activation. It is possible that LHX3 expression during pituitary development results in LHX3-INHAT interactions that function to prevent gene repression by INHAT. LHX3 may therefore work both indirectly by disrupting core INHAT proteins in a “de-repression” function, and directly by serving as a DNA-binding transcription factor on pituitary target genes. In this model, INHAT association with promoter DNA, through histone-binding and potentially other unknown co-factor(s), facilitates gene silencing. Upon LHX3 expression, and possible association with the CBP global co-activator, interaction with the acidic domains of TAF-1β and LANP by the LHX3 C-terminus may disrupt the INHAT complex and thus cause its dissociation from promoter chromatin. This INHAT dissociation would then allow for subsequent *trans*-activation of the target by LHX3 and CBP.

The experiments described here have identified INHAT components that are broadly expressed. In this context, LHX3 therefore could act as a specificity factor in the expression of pituitary tissue-specific genes with its functions determined by the chromatin microenvironment. However, we also hypothesize that the LHX3 C-terminus and other parts of the protein may interact with co-factor proteins that contribute to cell-specific functions of LHX3 during development.

Another possible mechanism may involve direct regulation of LHX3 by INHAT factors. Other proteins associated with chromatin remodeling and regulation have been shown to directly regulate transcription factors. In the heart, HOPX and HDAC2 interact to modulate GATA4 acetylation [68]. It is not known if LHX3 is acetylated, but it is also possible that the INHAT complex may mask LHX3 acetylation and therefore block activation of pituitary genes. Further experiments need to be performed in order to test these models.

We have described novel protein partners of the LHX3 regulatory protein that is critical for the development of both nervous and endocrine system tissues. Because mutations affecting the regions of the LHX3 protein involved in these interactions are associated with severe pediatric human diseases, these observations may have relevance to our understanding of the aberrant mechanisms underlying such diseases and may provide insights into the etiology of human endocrine diseases and allow future therapies and genetic counseling.
